# Genome-Wide Association Study Identifies QTNs and Candidate Genes Conferring Resistance to Soybean Frogeye Leaf Spot Race 7

**DOI:** 10.3390/plants15142106

**Published:** 2026-07-08

**Authors:** Yanzuo Liu, Bo Hu, Tianqi Xing, Pengfei Xu, Shuzhen Zhang, Wen-Xia Li, Hailong Ning

**Affiliations:** Key Laboratory of Soybean Biology, Ministry of Education, Northeast Agricultural University, Harbin 150030, China; 15645405369@163.com (Y.L.); hb554512341@163.com (B.H.); 15645500923@139.com (T.X.); xupengfei@neau.edu.cn (P.X.); zhangshuzhen@neau.edu.cn (S.Z.)

**Keywords:** soybean, frogeye leaf spot, 3VmrMLM model, QTN, LD blocks, resistance gene

## Abstract

Soybean (*Glycine max*) is a major economic and food crop whose yield is severely affected by frogeye leaf spot (FLS), caused by *Cercospora sojina*. Current knowledge of resistance genes remains insufficient for effective molecular breeding. In this study, a recombinant inbred line (RIL) population derived from a cross between the resistant parent, Henong 60 (H60), and the susceptible parent, Dongnong L13 (DN L13), was evaluated under field conditions in Acheng (AC) and Xiangyang (XY). Plants were artificially inoculated with physiological race 7 of *C. sojina*, and disease severity at the R3 growth stage was recorded. Genotyping using the SoySNP660K chip yielded 54,836 high-quality single-nucleotide polymorphism (SNP) markers. A genome-wide association study (GWAS) was performed using the 3VmrMLM model by integrating dual-environment phenotypic data, and four quantitative trait nucleotides (QTNs) significantly associated with resistance to FLS were identified on chromosomes 8 (1), 17 (1), and 20 (2). By the analysis of genomic annotation, functional enrichment, metabolic pathway analyses, haplotype–phenotype association and quantitative real-time PCR (qRT-PCR), *Glyma.20G155700* and *Glyma.17G070500* are intended to be candidate genes related to soybean resistance to race 7 of FLS. The findings of this study provide insights into the genetic mechanisms underlying resistance to FLS in soybean. The identified molecular markers and candidate genes may provide useful resources for marker-assisted breeding and the development of disease-resistant germplasm.

## 1. Introduction

Soybean frogeye leaf spot (FLS), also known as gray spot disease, is a foliar disease characterized by frog-eye-shaped lesions on leaves. The disease can reduce soybean yield by 10–60% and significantly decrease seed protein and fat content, thereby threatening both production and commercial value [1,2].

The causal agent of FLS, *Cercospora sojina* Hara, exhibits high genetic diversity and distinct physiological race differentiation. Kim et al. reported that sexual reproduction in this pathogen increases genetic variation and continuously generates new pathogenic races [3]. To date, multiple physiological races have been identified in major soybean-producing regions worldwide [4,5]. In China, races 1 and 7 are predominant, with race 7 being particularly widespread, highly virulent, and persistent, posing a serious threat to soybean production [6]. Therefore, identifying and deploying resistance genes effective against race 7 is important for developing durable FLS-resistant soybean cultivars.

Current strategies for FLS management include agronomic practices, biological control, and the development of disease-resistant varieties. Among these, breeding resistant cultivars is considered the most economical, environmentally friendly, and effective approach [7]. Traditional breeding efforts have identified several major resistance genes through phenotypic selection, such as *Rcs1* for race 1 and *Rcs2* for race 2 [8,9]. Advancements in molecular biology have significantly improved the efficiency of identifying disease-resistance genes through marker-assisted selection (MAS). For instance, Mian et al. mapped the FLS resistance gene *Rcs3* to linkage group J using simple sequence repeat (SSR) markers and identified closely linked markers Satt244 and Satt547 [10]. McAllister et al. employed single-nucleotide polymorphism (SNP) markers to identify two quantitative trait loci (QTLs) associated with FLS resistance on chromosomes 9 and 13, providing new targets for resistance gene discovery [7]. However, linkage-based mapping relies on biparental populations, which limits mapping accuracy and complicates the systematic identification of minor genes.

Genome-wide association studies (GWAS) utilize linkage disequilibrium (LD) in natural populations to identify high-resolution loci across the entire genome, eliminating the need to construct specific mapping populations. This approach is particularly useful for dissecting complex traits [11,12,13,14,15,16]. In recent years, GWAS has been applied to identify genes conferring resistance to *C. sojina* Hara in soybean. Sun et al. analyzed 183 soybean accessions and generated 23,156 high-quality SNPs using specific-locus amplified fragment sequencing (SLAF-seq). Using a compressed mixed linear model (CMLM), they identified four genes associated with resistance against race 1 of *C. sojina* Hara [17]. Na et al. evaluated 335 accessions and mapped 15 quantitative trait nucleotides (QTNs), identifying four candidate genes associated with resistance to race 7 [18]. Despite these advances, resistance to race 7 remains insufficiently characterized, and most candidate genes still lack functional validation.

In this study, we investigated a soybean recombinant inbred line (RIL) population (RIL6013) consisting of 139 lines. Genotyping was performed using the SoySNP660K BeadChip, yielding 54,836 high-quality SNP markers. GWAS was conducted using the 3VmrMLM model combined with multi-environment phenotypic data collected from two locations (AC and XY). LD block analysis based on four significant QTN loci identified four LD block regions containing 82 candidate genes. Sequence variation analysis between parental lines, haplotype analysis, and expression profiling following pathogen inoculation identified two genes strongly associated with resistance to physiological race 7 of *C. sojina* Hara. These findings improve the understanding of the genetic mechanisms underlying soybean FLS resistance and provide valuable resources for molecular marker-assisted breeding of resistant cultivars.

## 2. Materials and Methods

### 2.1. Genetic Materials

The RIL6013 population was developed by crossing the FLS-susceptible soybean cultivar DN L13 (susceptible parent) with the resistant cultivar H60 (resistant parent) to produce the F_1_ generation. The F_1_ plants were grown in alternating seasons from 2010 to 2014 in Harbin, Heilongjiang Province (126.63° E, 45.75° N), and Yazhou, Hainan Province (109.00° E, 17.50° N), and were self-pollinated for five consecutive generations using the single-seed descent method, yielding 139 homozygous recombinant inbred lines (RILs) [19]. The RIL6013 population was used for genome-wide association and haplotype analyses.

A second population, the germplasm resource (GP) panel, consisted of 455 high-quality soybean accessions, including 4 landraces, 387 Chinese cultivars, and 44 foreign cultivars [20] (Table S1). The GP panel was used for haplotype analysis.

### 2.2. Field Experiments

Field trials were conducted in 2022 at the AC experimental practice base (127.63° E, 45.82° N) and the XY experimental practice base (126.63° E, 45.75° N) of Northeast Agricultural University. The experimental material consisted of the RIL6013 population. Plants were grown at a density of 2.2 × 10^5^ plants/hm^2^. Nitrogen (N), phosphorus pentoxide (P_2_O_5_), and potassium oxide (K_2_O) were applied at rates of 18, 46, and 30 kg/hm^2^, respectively. The experiment followed a randomized complete block design with three replicates per environment. Each plot consisted of three ridges (3 m long and 0.67 m wide), with seeds sown in a single row at 0.07 m spacing. Irrigation, weed control, and pest and disease management were conducted according to standard local agricultural practices.

The meteorological factors such as the average temperature throughout the day (°C), the extreme maximum temperature throughout the day (°C), the extreme minimum temperature throughout the day (°C), the cumulative precipitation (mm), the total number of rainy days (d), the total sunshine duration throughout the day (h), and the average sunshine duration per day (h) during the period of bacterial infection (from 21 July to 10 August 2022) were retrieved from the Xiangfang National Meteorological Station (District station number: 50953) and the Acheng National Meteorological Station (District station number: 50958).

### 2.3. Preparation of Spore Suspension

Potato dextrose agar (PDA) medium was prepared, and the FLS pathogen *Cercospora sojina* physiological race 7 parental strain, stored at −4 °C, was retrieved. Under sterile conditions, a mycelial plug was excised using a sterile scalpel and transferred to a fresh PDA plate. Plates were sealed and incubated in the dark at 25 °C until the mycelium fully covered the medium surface. At this stage, the cultures were stored for further use.

Sorghum grains were cooked until the starch was exposed, then transferred to conical flasks, sealed, and sterilized by autoclaving. After cooling in a laminar flow hood, the preserved culture of the FLS physiological isolate race 7 was cut into small pieces and evenly inoculated onto the sorghum grains. Flasks were sealed and incubated at 25 °C in a biochemical incubator, with gentle shaking every 1–2 days to ensure uniform mycelial colonization.

Three days before inoculation, mycelium-covered sorghum grains were transferred to sterile gauze and misted daily with distilled water to maintain suitable humidity and temperature and induce asexual spore production. On the day of inoculation, spores were released by soaking the grains in distilled water and gently rubbing them through gauze to prepare a spore suspension. Debris was removed using a sieve, and the suspension was adjusted to a final concentration of 1 × 10^7^ conidia/mL. Sucrose was added to a final concentration of 3% (*w*/*v*) to enhance spore adhesion to soybean leaves.

The spore suspension was uniformly sprayed onto the leaves of soybean plants at the R3 growth stage. After inoculation, high humidity (>90%) was maintained to promote infection, and plants were kept under suitable temperature and light conditions until typical disease symptoms appeared.

### 2.4. Inoculation and Disease Evaluation

**Figure 1:**
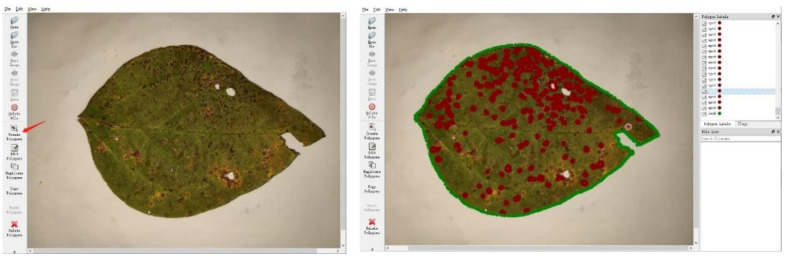
Annotation based on the original dataset. (before annotation on the left, after annotation on the right).

**Figure 2:**
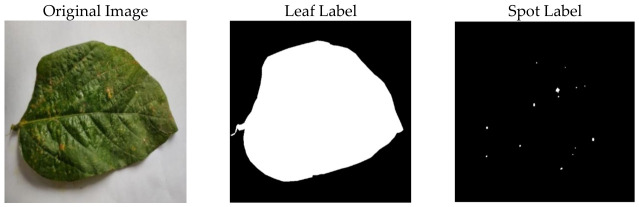
Original image labels acquired by threshold segmentation.

On 19 July 2022, plants of the RIL6013 population were inoculated with a *C. sojina* physiological race 7 conidial suspension (1 × 10^7^/mL). Twenty days after inoculation, four healthy individual plants were selected from each inoculated row to serve as four biological replicates; one fully expanded upper leaf with the most severe disease symptoms was harvested from each plant, yielding four diseased leaf samples per genotype. Considering that the disease severity of frogeye leaf spot varies greatly among different leaves within a single plant, random leaf selection may underestimate disease severity and weaken phenotypic differences among genotypes. To more accurately reflect the genetic resistance differences among lines, four leaves showing the most severe disease symptoms were uniformly selected from the whole plant without restricting leaf position. This phenotyping approach assesses the maximum disease susceptibility performance of each genotype rather than the average infection status of the whole plant. Consistent sampling criteria were applied to all experimental materials, which reduced evaluation randomness and avoided systematic bias in phenotypic identification. Leaves were photographed using a Canon DS126291 camera (Canon, Mumbai, India). Lesion and total leaf areas were quantified from the images using the image annotation tool LabelMe. The Create Polygons tool was used to annotate leaf lesions and whole leaves separately. Vertices were added along the boundaries of target leaf and lesion regions, and the last vertex was connected to the first vertex to form a closed area (Figure 1). Then the leaf labels and lesion labels were separately obtained using the threshold segmentation method (Figure 2). Lesion pixel counts and total leaf pixel counts were extracted, and the relative lesion area was calculated using the formula: relative diseased leaf area = lesion pixels/total leaf pixels.

Disease severity was scored on a 0–5 scale, with scores of 0 classified as resistant and scores of 3–5 classified as susceptible (Table 1).

### 2.5. Phenotype Variation Analysis

Phenotypic data for frogeye leaf spot disease severity in the RIL6013 population were analyzed following inoculation with race 7 of *Cercospora sojina Hara* at the AC and XY experimental sites. Descriptive statistics, including mean, coefficient of variation, kurtosis, and skewness, were calculated.

Analysis of variance (ANOVA) was performed on phenotypic data of relative spot area from three replicates across two environments. The ANOVA model included environment, genotype, and genotype × environment interaction effects. Genotype and genotype × environment interaction were treated as random effects to estimate genotypic variance, genotype × environment interaction variance, and error variance. Broad-sense heritability (*h*^2^) was then calculated using the formula:

(1) $$h^{2}=\frac{\sigma_{G}^{2}}{\sigma_{G}^{2}+\frac{\sigma_{GE}^{2}}{e}+\frac{\sigma_{\varepsilon }^{2}}{re}}$$

where *h*^2^ represents generalized heritability, $\sigma_{G}^{2}$ represents genotype variance, $\sigma_{GE}^{2}$ represents genotype-environment interaction variance, $\sigma_{\varepsilon }^{2}$ represents error variance, *e* represents the number of environments, and *r* represents the number of replicates.

All statistical analyses were conducted using SAS 9.2 software (SAS Institute, Cary, NC, USA).

### 2.6. Genome-Wide Association Analysis

High-quality genomic DNA was extracted from leaf tissue of the RIL6013 population using the CTAB method and genotyped with the SoySNP660K BeadChip (Beijing Boao Biotechnology Co., Ltd., Beijing, China). After quality filtering (minor allele frequency MAF ≥ 0.05, SNP missing rate < 10%, and exclusion of heterozygous loci), 54,836 high-quality SNP markers were retained and used in GWAS.

Phenotypic data were obtained by scoring lesions on a 0–5 scale following artificial inoculation with *Cercospora sojina* race 7 at the AC and XY experimental sites (Table S2). The 3VmrMLM model was applied to perform association analyses using single-environment phenotypic data to detect environment-specific quantitative trait nucleotides (QTNs). In addition, a joint analysis across the two environments was conducted to identify stably inherited loci [21]. MapChart was used to visualize the chromosomal distribution of the detected QTNs.

### 2.7. Candidate Gene Prediction

Linkage disequilibrium (LD) decay distance of the detected QTN was analyzed using TASSEL v5.0 (https://tassel.bitbucket.io/, accessed on 1 January 2023) with the negative natural logarithm method. The physical distance at which the r^2^ value declined to a half was used as the threshold for LD decay.

LD block analysis of FLS-resistance QTNs identified by genome-wide association analysis was implemented using Haploview v4.2 (https://sourceforge.net/projects/haploview/, accessed on 10 October 2025). For LD block analysis, each peak QTN SNP was used as the center with an analysis window of LD decay distance. LD blocks were defined using the confidence intervals method under default parameters, and the internal LD patterns were visualized via the built-in functions of Haploview.

Gene annotations strictly located inside the corresponding LD blocks were retrieved from Phytozome v13 (https://phytozome.jgi.doe.gov, accessed on 12 October 2025). The soybean (*Glycine max* Wm82.a2.v1) GFF3 genome file was adopted to collect gene names, physical positions, and functional descriptions within LD blocks.

Candidate genes were initially screened according to functional annotation. Genes annotated to participate in biotic stress response, plant immunity, and defense-related signaling pathways were retained, whereas genes associated with plant growth and development as well as constitutive housekeeping genes were excluded. Resequencing data of the RIL6013 parental lines (H60 and DN L13) were aligned and visualized using IGV software 2.19.8 (https://igv.org/, accessed on 10 October 2025) to screen inter-parental sequence variations. Genes carrying polymorphisms between two parents inside the LD blocks were determined as the final candidate genes.

### 2.8. Haplotype Analysis

Seventy-seven soybean plants with varying levels of disease susceptibility were selected from the RIL6013 recombinant inbred line population. Gene-specific primers were designed to amplify non-synonymous SNPs within the coding regions of candidate genes as well as SNP variants in their promoter regions (Table S3). Target fragments were amplified and sequenced. Lines carrying the genotype of the susceptible parent DN L13 were designated Hap-1, whereas those carrying the genotype of the resistant parent H60 were designated Hap-2. GraphPad Prism 10 (www.sciencesoftware.com.cn, accessed on 18 October 2025) was used to analyze haplotype–phenotype associations by integrating haplotype information with disease severity scores.

Whole-genome resequencing data from 455 soybean accessions were used to retrieve non-synonymous SNP data corresponding to the candidate genes. The GP population was categorized into haplotypes using the same criteria applied to the RIL6013 population. Haplotype–phenotype association analysis was then conducted using phenotypic data from 203 accessions (Table S4) to evaluate the universality of elite haplotypes across different genetic backgrounds.

In addition, 2883 soybean germplasm resources collected nationwide, including 1733 cultivated varieties, 1048 local varieties, and 102 wild accessions, were analyzed using resequencing data to determine the distribution of target gene haplotypes. The proportions of each haplotype were statistically compared among improved, local, and wild soybean groups.

### 2.9. Expression Pattern Analysis

To examine the temporal expression patterns of candidate genes harboring promoter base mutations and showing significant haplotype–phenotype associations following infection with *C. sojina* race 7, two parental soybean lines were used: H60, a resistant line carrying beneficial allelic variations in disease-resistant SNPs, and L13, a susceptible line lacking these beneficial alleles. These two lines differ markedly in their resistance to frogeye leaf spot.

Gene expression levels were analyzed at multiple time points after inoculation with *C. sojina* race 7 using quantitative real-time PCR (qRT-PCR). Leaf samples were collected at 0, 1, 3, and 6 h post-inoculation, with three biological replicates per genotype. Total RNA was extracted using the FastPure Universal Plant Total RNA Isolation Kit, and first-strand cDNA was synthesized using the HiScript III 1st Strand cDNA Synthesis Kit (+gDNA wiper). qRT-PCR was performed on a Roche LightCycler^®^ 480 real-time PCR system (Roche, Shanghai, China) using the SuperReal PreMix Plus kit, with three technical replicates per sample.

The *GmTUA5* gene was selected as the internal reference gene, and relative gene expression levels were normalized using the 2^−ΔΔCt^ method. Since expression data did not satisfy the prerequisites for parametric analysis, a nonparametric *t*-test (Mann–Whitney U test, GraphPad Prism) was exclusively used to evaluate significant differences in gene expression between genotypes at each inoculation time point.

Statistical analyses of gene expression differences across time points were conducted using GraphPad Prism 10 (http://www.graphpad-prism.cn, accessed on 18 October 2025) to assess the relationship between gene expression dynamics and disease-resistance phenotypes. Primer sequences used for qRT-PCR are listed in Table S5.

### 2.10. Protein Tertiary Structure Prediction

For candidate genes exhibiting non-synonymous SNP sites in the coding region and showing significant haplotype–phenotype associations, protein tertiary structures were predicted. Coding sequences corresponding to Hap-1 and Hap-2 were translated using the NovoPro tool (https://www.novopro.cn/tools/translate.html, accessed on 28 October 2025). Protein tertiary structures were predicted using AlphaFold3 (https://alphafoldserver.com/, accessed on 28 October 2025) with default parameters, and the model with the highest pLDDT score was selected. Structural differences between haplotypes were visualized using PyMOL (https://pymol.org/, accessed on 28 October 2025) to evaluate the potential effects of amino acid substitutions and tertiary structural variation on protein function.

## 3. Results

### 3.1. Descriptive Statistics and Analysis of Variance for Relative Lesion Area

Statistical analysis of meteorological factors in AC and XY from 21 July to 10 August 2022 revealed that the two locations had very similar temperatures. However, AC experienced more rainy days and higher cumulative precipitation than XY, while AC recorded fewer total sunshine hours and lower average daily sunshine duration compared to XY (Table 2).

The RIL6013 population was evaluated for FLS resistance in two environments (XY and AC) following inoculation with *C. sojina* physiological race 7. Disease severity was scored on a 0–5 scale, and descriptive statistics were calculated (Table 3). The mean disease severity score was 1.34 (SD = 1.731) in the XY environment and 1.73 (SD = 1.022) in the AC environment, indicating slightly higher disease severity in the AC environment. Phenotypic variation was greater in XY, as reflected by the higher standard deviation, suggesting greater genetic diversity in resistance responses under this environment. In XY, the distribution was moderately right-skewed (skewness = 0.4593) with negative kurtosis (−0.5639), indicating a relatively uniform distribution. In contrast, the AC environment exhibited stronger right skewness (1.362) and positive kurtosis (2.512), suggesting that most plants exhibited low disease severity, while a small proportion were highly susceptible. Overall, the continuous distribution and broad phenotypic variation indicated that the population was suitable for genome-wide association analysis.

ANOVA and heritability estimates were conducted using phenotypic data from both environments (Table 4). Environmental effects were not significant, indicating that the environment alone had limited influence on disease severity. In contrast, genotypic variance was highly significant (*p* < 0.0001), demonstrating substantial genetic variation for FLS resistance within the population. The genotype × environment interaction effect was also highly significant (*p* < 0.0001), indicating differential responses of genotypes across environments. The broad-sense heritability of FLS resistance across environments was estimated at 26%, suggesting that phenotypic variation in the RIL6013 population was strongly influenced by genotype × environment interactions rather than genotype alone (Table 4).

### 3.2. QTN Underlying FLS Resistance

**Figure 3:**
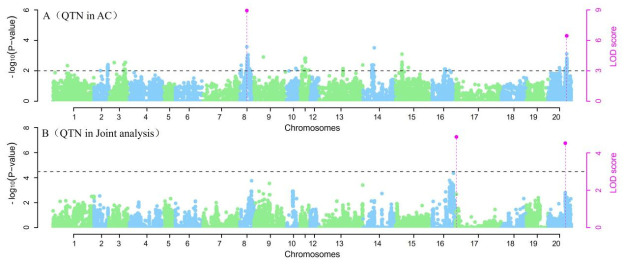
Manhattan plots of genome-wide association analysis in the RIL6013 population. (**A**) QTNs mapped in the Acheng (AC) environment. (**B**) QTNs identified by the average values of the two environments. The dashed line is the threshold for the significant QTN selected in the first stage.

Genome-wide association analysis was performed using the 3VmrMLM model to identify quantitative trait nucleotides (QTNs) associated with FLS resistance in the RIL6013 population. In the AC environment, two significant QTNs (LOD ≥ 3) were detected: AX-157408433 on chromosome 8 (LOD = 8.9347, phenotypic contribution rate r^2^ = 22.2463%) and AX-157461692 on chromosome 20 (LOD = 6.4416, r^2^ = 11.6831%). Together, these loci accounted for 33.9% of the phenotypic variation (Figure 3). Among them, AX-157408433 showed the largest effect and was considered a major resistance locus in the AC environment.

No significant QTNs (LOD < 3) were detected in the XY environment alone. However, joint analysis across both environments identified two additional QTNs: AX-157253184 on chromosome 17 (LOD = 4.8553, r^2^ = 6.3711%) and AX-157305217 on chromosome 20 (LOD = 4.5218, r^2^ = 4.8701%). These loci collectively explained 11.1% of the phenotypic variation and may represent environmentally responsive loci (Table 5). These results provide a basis for the subsequent identification of candidate genes.

### 3.3. Candidate Genes Related to FLS Resistance

In the LD decay analysis, at the physical distance of 200 kb, the r^2^ value declined to half, 0.2; thus, the LD decay distance was estimated as 200 kb.

LD analysis was conducted on regions extending 100 kb upstream and downstream of the four QTN identified through genome-wide association analysis to refine candidate intervals (Table 6). The LD block containing the AX-157305217 locus had five haplotypes with a cumulative frequency of 97.7%, while the block containing AX-157461692 comprised six haplotypes totaling 94.9%. The regions containing the AX-157408433 and AX-157253184 loci each had four haplotypes, with cumulative frequencies of 95.7% and 99.9%, respectively. These findings suggest that each LD block region was dominated by a few haplotypes, indicating low recombination and highlighting these regions as potential candidates for key functional genes (Figure S1).

All genes within the four LD blocks were retrieved from the soybean genome database in Phytozome, totaling 82 genes. Based on functional annotations provided by Phytozome, we preliminarily retained genes annotated to participate in biotic stress response, plant immunity, defense signal transduction and protein modification pathways and eliminated genes annotated for plant vegetative and reproductive growth, cell structure composition and constitutive housekeeping functions. In this way, 21 candidate genes were preliminarily screened out from the 82 genes. Among the 21 preliminary candidates, we further checked parental sequence variation and performed haplotype analysis (Table 7).

### 3.4. Haplotypes of Candidate Genes

Our study identified two haplotypes based on sequence differences in the candidate genes between the parental lines. Hap-1 corresponded to the susceptible parent DN L13, whereas Hap-2 corresponded to the disease-resistant parent H60 (Table 8). Specific primers were designed for the variant sites of the candidate genes. Genomic DNA was extracted from 77 RIL6013 lines showing significant phenotypic variation. The extracted DNA was used as a template for PCR amplification and sequencing of the target genes.

**Figure 4:**
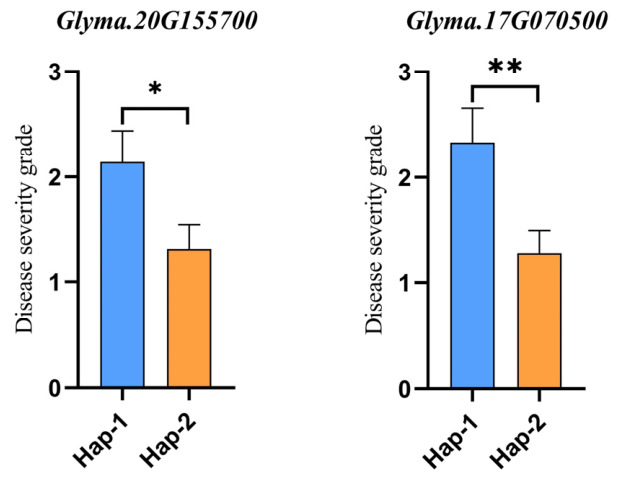
Haplotype–phenotype association analysis of *Glyma.20G155700* and *Glyma.17G070500* in the RIL6013 population. Note: The bar height denotes the average disease severity grade. Different colors distinguish sample groups. Hap-1 and Hap-2 represent distinct gene haplotypes. A nonparametric *t*-test (Mann–Whitney U test, GraphPad Prism) was used for intergroup comparisons, as phenotypic data did not conform to normal distribution; * represents *p* < 0.05, ** represents *p* < 0.01. For *Glyma.20G155700*, the number of Hap-1 and 42 as Hap-2 were 35 and 42, respectively, while for *Glyma.17G070500*, the number of Hap-1 and 42 as Hap-2 were 30 and 47, respectively.

Based on the sequencing results, haplotypes were assigned for each candidate gene. For *Glyma.20G155700*, 35 samples were classified as Hap-1 and 42 as Hap-2, while for *Glyma.17G070500*, 30 samples were Hap-1 and 47 were Hap-2 (Table S6). Haplotype–phenotype association analysis was conducted using disease severity grade. Since the phenotypic data failed to satisfy normal distribution assumptions, a nonparametric *t*-test (Mann–Whitney U test) embedded in GraphPad Prism was applied to compare Hap-1 and Hap-2 groups for both genes. The results showed that Hap-2 samples for both candidate genes had significantly lower disease severity grades than Hap-1 samples. These findings indicate that the Hap-2 haplotypes of *Glyma.20G155700* and *Glyma.17G070500* are associated with resistance to frogeye leaf spot (Figure 4).

**Figure 5:**
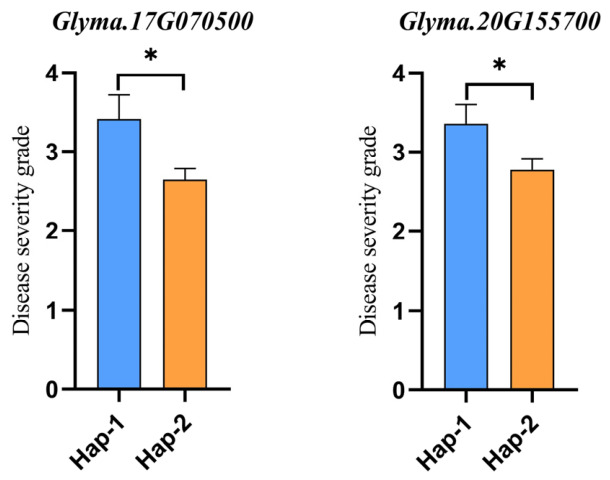
Haplotype–phenotype association analysis of *Glyma.20G155700* and *Glyma.17G070500* in the GP population. Note: The bar height denotes the average disease severity grade. Different colors distinguish sample groups. Hap-1 and Hap-2 represent distinct gene haplotypes. A nonparametric *t*-test (Mann–Whitney U test, GraphPad Prism) was used to assess intergroup differences, with * representing *p* < 0.05.

To further evaluate the effects of the candidate genes on FLS resistance across different genetic backgrounds, haplotype–phenotype association analysis of the two candidate genes was conducted in additional populations. The analysis utilized resequencing data from the germplasm resource (GP) population and disease susceptibility grade phenotypic data from 203 samples. In the GP population, 147 accessions were categorized as Hap-1 and 41 as Hap-2 for *Glyma.20G155700*, whereas 156 accessions belonged to Hap-1 and 28 to Hap-2 for *Glyma.17G070500*. As the phenotypic data failed to meet the criteria for normal distribution, a nonparametric *t*-test (Mann–Whitney U test) implemented in GraphPad Prism was adopted to conduct pairwise comparisons between the haplotype groups of both genes. The haplotype–phenotype association analysis revealed that samples carrying the Hap-2 haplotype for both genes exhibited significantly lower disease susceptibility grades than those carrying Hap-1. Importantly, the Hap-2 haplotypes were consistently associated with resistance in the GP population, consistent with the results observed in the RIL6013 population (Figure 5). Overall, these results suggest that *Glyma.20G155700* and *Glyma.17G070500* are associated with resistance to soybean FLS caused by *C. sojina* physiological race 7 across different genetic backgrounds.

**Figure 6:**
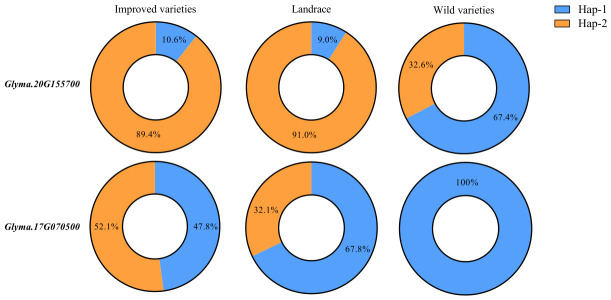
Distribution of haplotypes for candidate genes across soybean germplasm groups.

Using resequencing data from 2883 soybean accessions in the China National Center for Bioinformation (https://ngdc.cncb.ac.cn/soyomics/index, accessed on 1 January 2023), we analyzed the distribution of haplotypes for the candidate genes *Glyma.20G155700* and *Glyma.17G070500* across wild, landrace, and improved soybean varieties. The superior haplotype Hap-2 of *Glyma.20G155700* was detected in 89.4% of improved varieties and 91% of landraces, but only 32.6% of wild accessions (Figure 6), suggesting possible selection during early domestication. In contrast, the Hap-2 haplotype of *Glyma.17G070500* was present in 52.1% of improved varieties and 32.1% of landraces but was absent in wild accessions (Figure 6), indicating that this beneficial haplotype may have been selected during modern soybean breeding.

### 3.5. Expression Analysis of Candidate Genes After Inoculation

**Figure 7:**
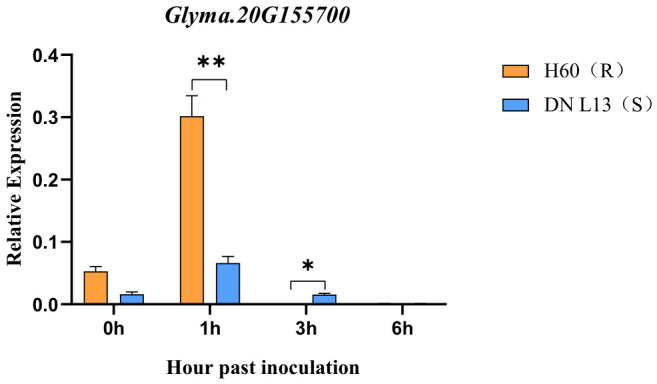
Expression patterns of *Glyma.20G155700* following inoculation with *Cercospora sojina* race 7. Note: Bar height denotes the relative mRNA expression level in resistant parent H60 and susceptible parent DN L13. Values represent the mean of three independent biological replicates. A separate nonparametric *t*-test (Mann–Whitney U test, GraphPad Prism) was applied to compare H60 and DN L13 at every inoculation time point, as expression data did not conform to normal distribution; * and ** indicates the difference is significant at the level of 0.05 and 0.01, respectively.

The relative expression levels of the candidate gene *Glyma.20G155700*, which harbors promoter-region mutations and beneficial haplotypes, were examined in the resistant parent H60 and susceptible parent DN L13 following inoculation with *C. sojina* race 7. At each single time point (0 h, 1 h, 3 h, 6 h post-inoculation), an independent pairwise comparison between H60 and DN L13 was performed. Since expression data failed the normal distribution test, a nonparametric *t*-test (Mann–Whitney U test, GraphPad Prism) was applied for separate comparison at every time point. The results showed that *Glyma.20G155700* expression initially increased following inoculation and then declined over time. No significant difference in expression was observed between the two varieties at 0 h post-inoculation. Expression peaked at 1 h post-inoculation, with significantly higher transcript levels in H60 than in DN L13 (*p* < 0.01). Thereafter, expression gradually decreased and reached very low levels in both varieties by 6 h post-inoculation. These findings suggest that *Glyma.20G155700* may positively regulate soybean resistance to physiological race 7 causing FLS disease (Figure 7).

**Figure 8:**
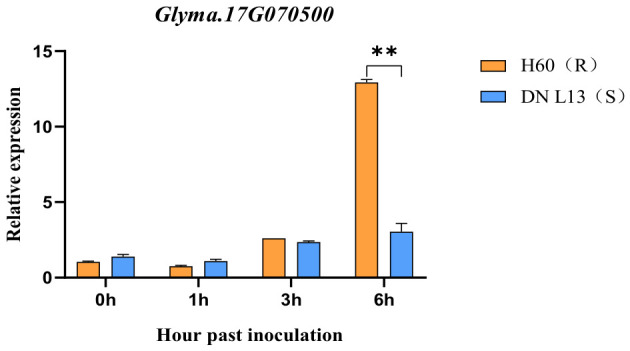
Expression patterns of *Glyma.17G070500* following inoculation with *Cercospora sojina* race 7. Note: Bar height denotes the relative mRNA expression level in resistant parent H60 and susceptible parent DN L13. Values represent the mean of three independent biological replicates. A separate nonparametric *t*-test (Mann–Whitney U test, GraphPad Prism) was applied to compare H60 and DN L13 at every inoculation time point, as expression data did not conform to normal distribution; ** indicates *p* < 0.01.

The expression pattern of *Glyma.17G070500*, a candidate gene carrying a non-synonymous mutation in its exon, was further examined in the resistant parent H60 and susceptible parent DN L13 following inoculation with *Cercospora sojina* race 7. At each single time point (0 h, 1 h, 3 h, 6 h post-inoculation), an independent pairwise comparison between H60 and DN L13 was carried out. Since qRT-PCR expression data failed the normal distribution test, a nonparametric *t*-test (Mann–Whitney U test, GraphPad Prism) was adopted for separate comparison at each time point. Transcript levels of *Glyma.17G070500* exhibited a continuous increase after inoculation. No significant differences in expression were detected between the two parents at 0, 1, or 3 h post-inoculation. Expression peaked at 6 h post-inoculation, with significantly higher transcript abundance in H60 than in DN L13 (*p* < 0.01). These findings suggest that *Glyma.17G070500* may positively regulate soybean resistance to race 7 of frogeye leaf spot (Figure 8).

### 3.6. Prediction of Protein Tertiary Structure

**Figure 9:**
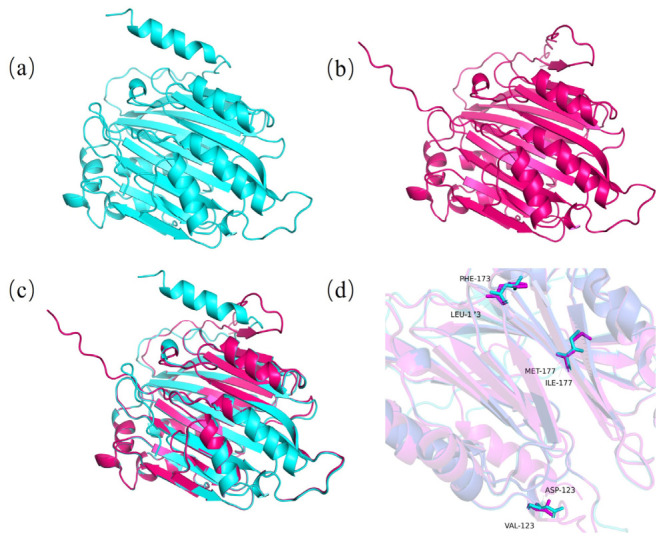
Predicted tertiary structures of *Glyma.17G070500* protein haplotypes and their structural comparison. (**a**) Tertiary structure of the Hap-1 protein. (**b**) Tertiary structure of the Hap-2 protein. (**c**) Superimposed tertiary structures of the Hap-1 and Hap-2 proteins. (**d**) Binding sites influencing the tertiary structure of the protein.

Three non-synonymous mutations in the exon of *Glyma.17G070500* resulted in the following amino acid substitutions: aspartic acid at position 123 to valine, leucine at position 173 to phenylalanine, and isoleucine at position 177 to methionine. These substitutions have the potential to alter the protein tertiary structure, which may be associated with differences in protein function. Using AlphaFold3, the tertiary structures of the two haplotypes were predicted. The model with the highest predicted local distance difference test (pLDDT) score was selected as the representative structure. Visualization was performed using PyMOL, and the root-mean-square deviation (RMSD) of Cα atoms was calculated to be 6.09 Å, indicating a notable divergence in the predicted spatial structures of the two proteins (Figure 9).

## 4. Discussion

### 4.1. The Influence of Meteorological Factors on the Occurrence of C. sojina in Two Environments

Based on previous studies, the meteorological factor thresholds for frogeye leaf spot (*Cercospora sojina*) in soybean are summarized as follows: the minimum lower temperature threshold for infection is 15 °C, and 48 h of leaf wetness is required [22,23]; the optimal temperature for infection (sporulation) ranges from 25 °C to 28 °C [24]; the high-temperature inhibition threshold (where spore germination is impeded) is above 35 °C [25]. For the triggering of pathogen transmission (spore splash dispersal), the required daily rainfall is no less than 0.1 mm [22]; the precipitation threshold for a moderate epidemic is that cumulative precipitation within 21 days after flowering reaches at least 170 mm, with no fewer than 12 rainy days; the precipitation threshold for a severe epidemic is that cumulative precipitation is no less than 200 mm, with continuous rainy weather lasting for 7 days or more [26]. The high-risk threshold for low light conditions is that the average daily sunshine duration is less than 10 h, which extends the duration of leaf wetness retention [27]; the light inhibition threshold is that the average daily sunshine duration is no less than 11 h, which significantly reduces disease severity [23,25]. In this study, during the infection period of *Cercospora sojina* (from 21 July to 10 August 2022), the temperature in both XY and AC regions fell within the suitable range for soybean FLS. However, differences in the three key meteorological factors—total precipitation, sunshine duration and minimum temperature—led to significant differentiation in disease epidemic intensity between the two regions. In terms of temperature, the ten-day average temperature in AC was 0.6–1.0 °C higher than the overall average; there was no long-term low-temperature stress below 15 °C, resulting in a shorter pathogen incubation period and faster reproduction, while the phased low temperature in XY temporarily blocked the rapid expansion of the disease. The precipitation condition is the core driving factor for the difference: the cumulative precipitation in AC was 43.6 mm higher than that in XY, with frequent heavy rains, waterlogged soil and continuous high humidity on leaf surfaces, which provides optimal conditions for spore dispersal, germination and multiple rounds of reinfection. In contrast, although there were more rainy days in XY, the rainfall intensity was generally low, and field humidity decreased rapidly, thus limiting infection intensity. Light condition plays an auxiliary regulatory role: the average daily sunshine duration in XY is longer, which improves the field microclimate, shortens the water retention time on leaves, and reduces the risk of disease epidemic, while the low light condition in AC exacerbates the high humidity in the closed canopy and amplifies the pathogenic effect of rainfall.

### 4.2. Advantages and Disadvantages of Phenotypic Measurement Sampling Method

In the past, the evaluation of soybean gray spot disease resistance was mostly conducted using the susceptibility index method, which weighted and evaluated various susceptible leaf states in the plants [17,28]. In this study, samples were taken from the most severely affected leaves on the diseased plants for the measurement of relative lesion area and the classification of susceptibility levels. However, this approach characterizes the maximum susceptibility of the genotype, rather than the average degree of damage to the plant. Theoretically, this method reduces the differences in disease resistance among varieties. Practically, this method is conducive to screening out extreme resistant or susceptible varieties. However, it has the drawback of eliminating the medium-resistant varieties.

### 4.3. Evaluation of Phenotypic Variation and Genetic Characteristics of Resistance

Descriptive statistical analysis revealed that the RIL6013 population exhibits significant phenotypic variation in both XY and AC environments, with distinct distribution characteristics. In the AC environment, the population displayed a higher average disease grade and a smaller standard deviation, along with a right-skewed and peaked distribution. This suggests that most plants were relatively resistant, while a few were highly susceptible to the disease. In contrast, the XY environment exhibited a more uniform phenotypic distribution with a larger standard deviation (Table 3). Analysis of variance for both individual environments and combined conditions indicated highly significant genotypic variances (*p* < 0.0001) (Table 4), confirming a genetic basis for soybean resistance to physiological race 7 of *C. sojina*, which is essential for subsequent genome-wide association studies.

Joint analysis also revealed a highly significant genotype × environment (G × E) interaction (*p* < 0.0001), indicating that the expression of resistance varies among genotypes across environments. The broad-sense heritability was calculated to be 26%, suggesting that phenotypic variation in soybean resistance is primarily influenced by the interplay of genetic and environmental factors rather than by stable major genetic factors. These results are consistent with previous studies, which indicate that FLS, or gray spot disease, is a complex, multi-gene-controlled, and environment-sensitive quantitative trait [17,29,30].

In this study, the 3VmrMLM model was employed for both single-environment and multi-environment analyses, enabling the identification of stable and valuable resistance loci [31,32,33]. This approach effectively detects major-effect QTNs within a single environment while accounting for environmental interaction effects and has been successfully applied in analyses of various crop traits [34,35,36]. In this research, four environment-specific QTNs were identified: AX-157408433 and AX-157461692 in AC, and AX-157253184 and AX-157305217 in the combined analysis using 3VmrMLM. These loci provide a basis for further exploration of candidate genes.

### 4.4. Analysis of Different QTN Loci in a Polygenetic Background

Genome-wide association analysis of the RIL6013 population identified four QTN loci associated with soybean frogeye leaf spot resistance. Comparisons with the SoyBase database and published studies showed that two detected QTNs are distributed in genomic regions adjacent to previously reported disease-resistance intervals (Figure S2). Specifically, the physical distance between AX-157408433 and the resistance marker Satt233 for FLS race 1 reported by Wang et al. is 586 kb. AX-157461692 is located 830 kb and 850 kb away from the markers Afx-89163218 and Afx-89210591 for FLS race 15 identified by Gu et al. [28], respectively. Given that the estimated LD decay distance of the RIL6013 population is about 300 kb, the physical distances of 586–850 kb substantially exceed the range of close linkage, indicating that these loci are not tightly linked at the local LD level. Nevertheless, they reside within the same large conserved resistance-related genomic segment as the previously reported intervals. Although earlier linkage analyses tended to yield relatively coarse mapping intervals with low false-positive rates, the GWAS strategy adopted in the present study substantially refined and narrowed the target candidate regions. The genomic correspondence with published results provides additional genetic support for the reliability of the detected QTNs. The remaining two QTNs (AX-157253184 and AX-157305217) are distant from previously documented resistance loci and may represent novel candidate resistance loci, which require further verification in future studies.

### 4.5. Haplotype–Phenotype Association Analysis of Candidate Genes in a Polygenetic Background

This study provides genetic evidence supporting the functional relevance of candidate genes through haplotype analysis using the RIL6013 and GP populations. We identified 77 materials from the RIL6013 population exhibiting varying levels of susceptibility and conducted haplotype analysis of two candidate genes, *Glyma.20G155700* and *Glyma.17G070500*. Using the high homogeneity and relatively simple genetic background of the recombinant inbred population, two beneficial haplotypes strongly associated with disease-resistant phenotypes were identified.

In the RIL6013 population, the frequencies of beneficial haplotypes in disease-resistant materials (disease grade 0–2) were 0.6667 for *Glyma.20G155700* and 0.6595 for *Glyma.17G070500*. A subsequent haplotype analysis was performed using 203 accessions from the GP population. In this natural population, the frequencies of beneficial haplotypes in disease-resistant materials (disease grade 0–2) were 0.471 for *Glyma.17G070500* and 0.416 for *Glyma.20G155700*, indicating that beneficial haplotypes of these genes are also associated with resistance to FLS disease in diverse genetic backgrounds.

Notably, beneficial haplotypes of both genes were associated with stronger disease resistance across two distinct populations, pointing to comparatively stable correlative effects. In this study, disease-resistance genes were first localized using the high homogeneity and relatively simple genetic background of a recombinant inbred population [37]. Subsequently, superior haplotypes were identified in the natural population [38,39]. Collectively, these results provide genetic evidence that *Glyma.20G155700* and *Glyma.17G070500* may participate in the resistance response to physiological race 7 of *C. sojina*.

### 4.6. Screening of Candidate Genes

In this study, genome-wide association analysis was performed using the RIL6013 population, resulting in the identification of four QTN loci associated with resistance to FLS in soybean. SNPs located within 100 kb upstream and downstream of these QTN loci were screened for LD block analysis. Gene annotation information within each LD block was retrieved from the Phytozome database. Based on gene annotation, 21 candidate genes were initially predicted. By comparing sequence differences between parental lines, two genes were ultimately identified as potentially involved in resistance to physiological race 7 of *C. sojina* causing FLS disease.

The *Glyma.20G155700* gene is located within the LD block of the FLS resistance QTN locus AX-157461692 and is situated 830 kb and 840 kb away from SNP loci Afx-89163218 and Afx-89210591, respectively, which were previously reported to be associated with physiological race 15. This gene encodes an F-box protein (PP2-A14), which has been documented to participate in plant immune processes. As a substrate recognition subunit of the SCF ubiquitin ligase complex, F-box proteins mediate the ubiquitination and degradation of target proteins involved in diverse biological processes [40,41]. In plant stress resistance, F-box proteins activate the jasmonic acid/ethylene signaling pathway by targeting disease-resistance regulatory factors for degradation, thereby promoting the expression of defense-related proteins [42]. Additionally, they can directly recognize and degrade pathogenic effector proteins, further strengthening plant defense capacity [43,44,45,46].

In this study, sequence variations were detected in the promoter region of *Glyma.20G155700* between the parental lines, which may influence transcriptional activity. Subsequent qRT-PCR analysis showed that *Glyma.20G155700* expression was upregulated after inoculation with *C. sojina* race 7, with higher expression levels observed in the resistant parent. These results suggest that *Glyma.20G155700* may participate in soybean defense against FLS.

The *Glyma.17G070500* gene is located within the LD block of QTN locus AX-157305217, which is associated with resistance to FLS. This gene encodes S-adenosylmethionine decarboxylase (AdoMetDC), an enzyme that catalyzes the conversion of S-adenosylmethionine (SAM) to decarboxylated SAM, a key intermediate required for polyamine biosynthesis, including spermidine [47,48]. Polyamines, including putrescine and spermidine, play important roles in regulating plant cell proliferation, stress responses, and defense signal transduction. They inhibit pathogenic bacterial growth, maintain reactive oxygen species (ROS) homeostasis, and enhance resistance to external stressors through histone modification or activation of transcription factors [49,50,51].

Non-synonymous SNPs (nsSNPs) were identified in the coding region of *Glyma.17G070500* between parental lines, which may alter the spatial structure of the encoded protein. Tertiary structure predictions for the two genotypes revealed significant structural differences, further supporting the potential role of *Glyma.17G070500* as a candidate gene associated with soybean resistance to FLS.

The reference gene GmTUA5 is widely recognized as a reliable reference gene in soybean research. Studies have demonstrated that it maintains high expression stability under various abiotic and biotic stress conditions, including drought, salt stress, and viral infections. This stability makes GmTUA5 an appropriate choice for use as a reference gene in quantitative real-time PCR experiments [52,53].

Functional annotation, haplotype analysis, and qRT-PCR results collectively indicate that *Glyma.20G155700* and *Glyma.17G070500* respond to biological stress and may participate in soybean defense processes against frogeye leaf spot. Future studies employing RNA interference (RNAi) and CRISPR/Cas9 gene-editing technologies will further validate their functional roles, which will accelerate the utilization of these two genes as markers in molecular assistant selection.

## 5. Conclusions

In this study, the RIL6013 recombinant inbred population was used to identify QTNs associated with resistance to physiological race 7 of FLS in soybean. Two candidate genes, *Glyma.20G155700* and *Glyma.17G070500*, were identified as potentially contributing to resistance against FLS caused by *Cercospora sojina*. These findings provide candidate genes and a theoretical basis for the molecular breeding of FLS-resistant soybean cultivars. Future studies, including gene overexpression and knockout experiments, will be necessary to further validate the functions of these candidate genes. In addition, the development of molecular markers closely associated with these genes may facilitate the incorporation of FLS resistance into soybean molecular breeding programs.

## Figures and Tables

**Table 1:** Disease severity classification based on relative lesion area.

Scale	Resistance-Susceptibility Degree	Relative Spot Area (%)
0	Highly resistant	0–1%
1	Resistant	1–3%
2	Moderately resistant	3–6%
3	Moderately susceptible	6–12%
4	Susceptible	12–25%
5	Highly susceptible	>25%

**Table 2:** Statistical analysis of meteorological factors in Xiangyang and Acheng from 20 July to 10 August 2022.

Statistical Index	Temperature			Precipitation		Sunshine Duration	
	Average (℃)	Maximum (℃)	Minimum (℃)	Cumulative Precipitation (mm)	Total Number of Rainy Days (d)	Total Daily Sunshine (h)	Daily Average Sunshine Duration (h)
XY	24	34	14.2	175.2	13	222.5	10.6
AC	24.7	33	15	218.8	11	205.7	9.79

**Table 3:** Descriptive statistics of FLS scores in the RIL6013soybean population across two environments.

Environment	Dongnong L13	Henong 60	Average	Standard Deviation	Minimum	Maximum	Skewness	Kurtosis
XY	5	0	1.34	1.731	0	5	0.4593	−0.5639
AC	5	1	1.73	1.022	0	5	1.362	2.512

**Table 4:** Analysis of variance (ANOVA) and heritability estimates for relative spot area of FLS in the RIL6013 population across environments.

Environment	Source	DF	SS	MS	F	Pr > F	Variance
XY	Replications	2	0.00000873	0.00000437	0.01	0.9927	
	Genotypes	137	0.42558674	0.00310647	5.23	<0.0001	0.00084
	Error	274	0.16262812	0.00059353			0.00059
	Total	413	0.5882236				
	h^2^						0.58520
AC	Replications	2	0.00066372	0.00033186	0.35	0.7021	
	Genotypes	136	0.63821229	0.00469274	5.01	<0.0001	0.00125
	Error	272	0.25485686	0.00093697			0.00094
	Total	410	0.89373287				
	h^2^						0.57195
Joint	Environments	1	0.00013012	0.00013012	0.17	0.6801	
	Environment (Replication)	4	0.00067246	0.00016811	0.22	0.9274	
	Genotypes	138	0.61645492	0.00446706	5.84	<0.0001	0.00020
	Environments × Genotypes	135	0.44734412	0.00331366	4.33	<0.0001	0.00085
	Error	546	0.41748498	0.00076462			0.00077
	Total	824	1.48207177				
	h^2^						26%

**Table 5:** Detailed information on QTNs associated with frogeye leaf spot resistance.

Environment	Marker	Chromosome	Position (bp)	LOD (Q)	Add	r^2^ (%)	*p*-Value
AC	AX-157408433	8	16,711,707	8.9347	−0.5112	22.2463	1.41395 × 10^−10^
AC	AX-157461692	20	39,429,867	6.4416	0.4247	11.6831	5.13948 × 10^−8^
Joint	AX-157253184	17	5,639,017	4.8553	−0.3202	6.3711	2.26168 × 10^−6^
Joint	AX-157305217	20	36,734,529	4.5218	0.3086	4.8701	5.03812 × 10^−6^

**Table 6:** Linkage disequilibrium (LD) block intervals and haplotype frequencies.

Marker	Chromosome	LD Block Interval	Proportion of Different Haplotypes (%)					
			H1	H2	H3	H4	H5	H6
AX-157305217	20	109 kb (36,654,402~36,763,858)	74.6	17.4	2.9	1.4	1.4	
AX-157408433	8	129 kb (16,582,497~16,711,707)	55.4	34.5	3.6	2.2		
AX-157461692	20	163 kb (39,412,952~39,576,517)	65.9	16.7	5.1	3.6	2.2	1.4
AX-157253184	17	145 kb (5,493,175~5,639,017)	68.8	18.8	10.9	1.4		

**Table 7:** Functional annotation of candidate genes.

Genes	Location	Annotation
*Glyma.20G123400*	Chr20:36606303..36608942	MATE efflux family protein-related
*Glyma.20G123500*	Chr20:36610054..36614582	MATE efflux family protein-related
*Glyma.20G124100*	Chr20:36661567..36669834	Tetratricopeptide repeat (TPR_2)/Tetratricopeptide repeat protein 5 OB fold domain
*Glyma.20G124700*	Chr20:36705435..36711795	Thiol oxidase/Sulfhydryl oxidase
*Glyma.20G124800*	Chr20:36713100..36715825	Membrane-associated RING finger protein
*G* *lyma.20G155500*	Chr20:39433274..39435445	MATE efflux family protein
*Glyma.20G155700*	Chr20:39454164..39457687	F-box protein PP2-A14
*Glyma.20G156600*	Chr20:39560200..39565397	E3 ubiquitin-protein ligase XBAT33
*Glyma.20G156700*	Chr20:39589071..39593170	Leucine-rich repeat (LRR_1)/Protein tyrosine kinase (Pkinase_Tyr)/Leucine-rich repeat N-terminal domain (LRRNT_2)/Leucine-rich repeat (LRR_8)
*Glyma.20G156800*	Chr20:39597728..39599181	Heat stress transcription factor B-2A
*Glyma.08G204700*	Chr08:16594092..16600507	Homeobox-leucine zipper protein GLABRA 2
*Glyma.08G204900*	Chr08:16602807..16605982	PRONE (plant-specific ROP nucleotide exchanger)
*Glyma.08G205100*	Chr08:16610100..16611569	Non-specific serine/threonine protein kinase/Threonine-specific protein kinase
*Glyma.08G206300*	Chr08:16682688..16685575	Regulator of VPS4
*Glyma.08G206400*	Chr08:16686954..16690055	Late embryogenesis abundant hydroxyproline-rich glycoprotein
*Glyma.17G070100*	Chr17:5493050..5494335	Aspartyl protease family protein
*Glyma.17G070300*	Chr17:5503555..5509036	Protein kinase domain (Pkinase)/Leucine-rich repeat N-terminal domain (LRRNT_2)/Leucine-rich repeat (LRR_8)
*Glyma.17G070500*	Chr17:5524596..5526914	Adenosylmethionine decarboxylase/S-adenosyl-L-methionine decarboxylase
*Glyma.17G070800*	Chr17:5546050..5549115	AP2 domain (AP2)
*Glyma.17G071700*	Chr17:5612498..5615941	Leucine-rich repeat (LRR_1)/Protein tyrosine kinase (Pkinase_Tyr)/Leucine-rich repeat N-terminal domain
*Glyma.17G071900*	Chr17:5625891..5629078	Calcineurin B subunit (protein phosphatase 2B regulatory subunit)-like protein

**Table 8:** Candidate genes with allelic differences between parental lines.

ID	Gene	Parents	Type	Position/bp (Promoter)	Position/bp (CDS)		
1				39,459,086			
	*Glyma.20G155700*	L13	Hap-1	C			
		H60	Hap-2	G			
2					5,526,050	5,526,201	5,526,213
	*Glyma.17G070500*	L13	Hap-1		A (D)	G (L)	T (I)
		H60	Hap-2		G (V)	C (F)	G (M)

## Data Availability

All data generated in this study are presented in the main text and Supplementary Materials.

## Supplementary Materials

The following supporting information can be downloaded at: https://www.mdpi.com/article/10.3390/plants15142106/s1, Table S1: Names of 455 soybean germplasm accessions; Table S2: Disease grade of RIL6013 population; Table S3: Quantitative primer sequences; Table S4: Disease grade of soybean accessions with different haplotypes of *Glyma.20G155700* and *Glyma.17G070500*; Table S5: Real-time PCR primer sequences for *Glyma.20G155700*; Table S6: Phenotypes of different haplotypes of *Glyma.20G155700* and *Glyma.17G070500*; Figure S1: Linkage disequilibrium (LD) haplotype block patterns around four FLS resistance QTNs in the RIL6013 population analyzed by Haploview v4.2; Figure S2: Chromosome mapping of four frogeye leaf spot resistance QTNs detected via GWAS in the RIL6013 population, with comparisons to previously reported flanking markers and candidate genes.
