# Supplementary material for: Genome-Wide Association Study Identifies QTNs and Candidate Genes Conferring Resistance to Soybean Frogeye Leaf Spot Race 7
Source: Plants (Basel). 2026 Jul 8;15(14):2106. doi: 10.3390/plants15142106 (PMC13414527; doi:10.3390/plants15142106)
Supplement: Supplementary file 1 [file plants-15-02106-s001.zip › Figure S1.pdf]

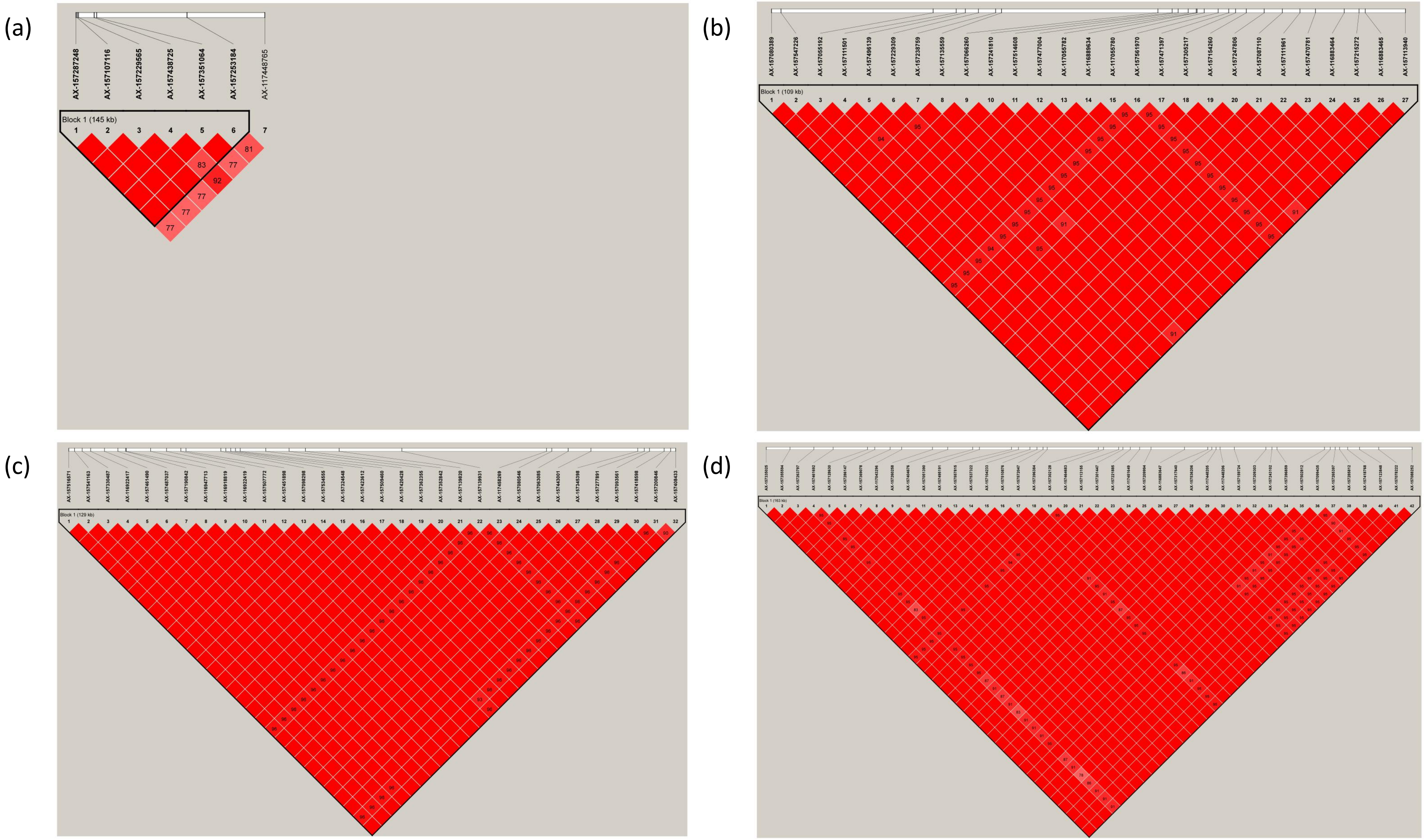

Figure S1. Linkage discquilibrium (LD) block haplotype paterns around four FLS resistance QINs in the RL6013 population analyzcd byHaploview v4.2. (a-d) Haplotype block structures corresponding to QINs AX-157253184, AX-157305217, AX-157408433, and AX157461692, respectively. Pairwise LD between SNPs is quantified by the squared allele frequency correlation (r^2)
