# Supplementary material for: Genome-Wide Association Study Identifies QTNs and Candidate Genes Conferring Resistance to Soybean Frogeye Leaf Spot Race 7
Source: Plants (Basel). 2026 Jul 8;15(14):2106. doi: 10.3390/plants15142106 (PMC13414527; doi:10.3390/plants15142106)
Supplement: Supplementary file 1 [file plants-15-02106-s001.zip › Table S1.pdf]

Table S1 – Names of 455 soybean germplasm accessions.

Cultivar name					
15-332	16QH173-2	Hefeng 22	Beidou 16	Dongnong 24	Acana
Am2330	Amuer	Hefeng 25	Beidou 23	Dongnong 36	Alvor
Am2345	Amuer 262	Hefeng 29	Beidou 40	Dongnong 42	Am2360
Am2376	Amuer 284	Hefeng 30	Beidou 42	Dongnong 43	Am2413
Am2384	Amuer 310	Hefeng 35	Beidou 53	Dongnong 47	Amuer (south)
Am2394	Beidou 10	Hefeng 40	Beifeng 11	Dongnong 48	Arica
Am2431	Beidou 14	Hefeng 43	Dongnong 5	Dongnong 49	Baojiao12-4142
Amuer 41	Beidou 19	Hefeng 45	Ha03-3764	Dongnong 50	Baojiao12-5219
Beidou 3	Beidou 21	Hefeng 51	Ha05-5675	Dongnong 51	Baojiao12-6314
Beidou 4	Beidou 34	Heinong 35	Ha06-1939	Dongnong 52	Baojiao13-5074
Beiyi 28	Beidou 41	Heinong 36	Hefeng 36	Dongnong 53	Baojiao13-5203
Beiyi 5	Beidou 42	Heinong 38	Hefeng 37	Dongnong 54	Baojiao13-6085
Beiyi 7	Beidou 47	Heinong 39	Hefeng 38	Dongnong 55	Baojiao13-7042
Beiyi 8	Beidou 49	Heinong 40	Hefeng 39	Dongnong 56	BD95
BH-2188	Beijiang 1	Heinong 41	Hefeng 41	Dongnong 57	BeijiangJiu 1
BH-2264	Beijiang 2	Heinong 47	Hefeng 44	Dongnong 60	BHNNCCM
BH-2353	BH-2232	Heinong 62	Hefeng 46	Dongnong 63	Caimengmaodou 5
Chidou 1	BH292-11	Heinong 69	Hefeng 47	Dongnong 64	Caimengmaodou 6
Daoulia	D98-335	Heinong 71	Hefeng 48	Dongnong 65	Cargolu blue
Dengke 1	E10135kn	Henong 69	Hefeng 49	Dongnong 66	Dongnongdou 245
Dengke 5	E10142kn	Henong 75	Hefeng 50	Dongnong 67	Dongsheng 8
E10136	E10143kn	Henong 76	Hefeng 54	Dongnong 69	E103k.n.A.R
Edou 1	E10kn	Hong14-24	Hefeng 56	Dongnong 70	E1066
Heihe 1	Fengshou 6	Kendou 30	Heinong 11	Dongnong 71	E10BH-189-9
Heihe 10	Habei46-1	Kendou 32	Heinong 19	Dongnong 72	E10BH204-09
Heihe 11	Hefeng 53	Kendou 34	Heinong 34	Dongqing 20	Excellent ratio
Heihe 12	Hefeng 55	Kendou 35	Heinong 37	Dongsheng 1	Gary
Heihe 14	Hefeng 57	Kendou 36	Heinong 43	Dongsheng 2	Heikang06-2
Heihe 15	Heihe 13	Kendou 38	Heinong 44	Dongsheng 6	Hong13-1809
Heihe 18	Heihe 17	Kendou 39	Heinong 48	Dongsheng 7	Huang Baozhu
Heihe 19	Heihe 24	Kendou 43	Heinong 49	Dongsheng 8	Jinchen1885
Heihe 20	Heihe 25	Kendou 47	Heinong 51	E1028k.n.r	K125
Heihe 21	Heihe 31	Kendou 48	Heinong 52	Fengshou 11	Kangxianchong 8
Heihe 22	Heihe 38	Kendou 52	Heinong 54	Fengshou 24	Kangxianchong 10
Heihe 23	Heihe 40	Kendou 54	Heinong 55	Fengshou 25	Kangxianchong 12
Heihe 27	Heihe 42	Kendou 56	Heinong 56	Harmonious	Kangxianchong 13
Heihe 28	Heihe 43	Kenfeng 10	Heinong 61	Hong14-623	Kangxianchong 5
Heihe 30	Heihe 44	Kenfeng 11	Heinong 64	Hong14-629	Kangxianchong 6
Heihe 33	Heihe 49	Kenfeng 13	Heinong 68	Hong14-688	Kenbao 11-427
Heihe 34	Heihe 51	Kenfeng 21	Heinong 8	Hongfeng 11	KenK11-6618
Heihe 35	Heihe 56	Kenfeng 23	Henong 59	Hualaidou 1	KenK11-7184
Heihe 36	Henong 63	Kenfeng 4	Henong 60	Jian13-780	ks-3
Heihe 37	Hu 489	Kenfeng 6	Henong 64	Jian13-824	KYD97
Heihe 39	Huajiang 1	Kenfeng 8	Henong 65	Jinchen 568	Ligia

Cultivar name					
Heihe 45	Huajiang 2	Nenfeng 16	Henong 66	Jingshanpu	Liujianlimaodou 1
Heihe 46	Huajiang 4	Nenfeng 18	Henong 67	Jinshangpu	Liujianlimaodou 2
Heihe 47	Jike 1	NongKen 1	Henong 68	Ken09-1870	Longdalaidou 1
Heihe 5	Jiuyan 19	Suinong 11	Jinong 18	Ken12-2117	Longdalaidou 2
Heihe 53	Jiuyan 2	Suinong 13	Jiunong 21	Ken13-1155	LongKen 3015
Heihe 54	Jiuyan 23	Suinong 14	Jiunong 29	Ken13-1514	LongKen 3021
Heihe 6	Jiuyan 4	Suinong 15	Kangxian 2	Kenfeng 18	LongKen 3203
Heihe 7	Jiuyan 6	Suinong 16	Kangxian 5	KenK10-367	Luzina Jirit
Heihe 8	Jiuyan 9	Suinong 17	Kangxian 9	Kenkedou 12	Mengdou 11
Jilin 35	Jiyu 69	Suinong 18	Kendou 26	Kenkedou 15	Mengdou 12
Jilin 39	Jiyu 87	Suinong 20	Kendou 28	Kenkedou 20	Mengdou 14
Jilin 40	Jiyu 88	Suinong 21	Kenfeng 14	Kenkedou 75	Mengdou 15
Jilin 48	Jiyu 95	Suinong 22	Kenfeng 15	Longken 323	Mengdou 16
Keshan 1	Kenfeng 16	Suinong 23	Kenfeng 17	Longken 330	Mengdou 20
Lcc-705	Kenkedou 1	Suinong 24	Kenfeng 18	Longken 342	Mengdou 28
Merlin	Kenkedou 4	Suinong 25	Kenfeng 20	Longken 345	Mengdou 30
MK-100	Kenkedou 5	Suinong 27	Kenfeng 22	Longken 348	Mengdou 36
Mufeng 1	Kenkedou 7	Suinong 28	Kenfeng 5	Longken 349	Mingxing 0901
Neidou 4	Kennong 63	Suinong 29	Kenjian 17	Longken 357	Nongda 35369
Nengao 1	Lcc339-04	Suinong 30	Kenjian 27	Longken 358	Quane 1
Nengao 2	Mengdou 9	Suinong 32	Kenjian 35	Longken 359	Shishengchangye
Nengao 3	N178	Suinong 33	Kenjian 43	Longken 381	Sonata
Nengao 4	Progres	Suinong 34	Kenjian 7	Longken 385	Suixiaolidou 1
Nengao 5	Shangdou 1	Suinong 35	Kennong 18	Longken 392	Suixiaolidou 2
Nengao 6	Silihuang	Suinong 36	Kennong 19	Longken 395	T01
Nengao 7	Silijia	Suinong 37	Kennong 22	Longken 397	Ulna
Pocanc l	Sky blue	Suinong 38	Kennong 26	Mancangjin	Zhenglvmaodou 1
Qinong 1	Suinong 26	Suinong 39	Kennong 28	Pengdou 158	Zhonghuang 68
Qinong 2	Xingnong 1	Suinong 4	Kennong 31	Polar bear	Zhongkemaodou 1
Qinong 3	Xingnong 2	Suinong 40	Kennong 4	Shengdou 15	Zhongkemaodou 4
Simina	Xingnong 3	Suinong 41	Nenfeng 15	Shiyueyi 70	Zihua 4
Sunset	Xingnong 4	Suinong 7	Nenfeng 4	Suiwuxing 2	
