# Supplementary figures and images for: Genome-Wide Association Study Identifies QTNs and Candidate Genes Conferring Resistance to Soybean Frogeye Leaf Spot Race 7
Source: Plants (Basel). 2026 Jul 8;15(14):2106. doi: 10.3390/plants15142106 (PMC13414527; doi:10.3390/plants15142106)

chr8

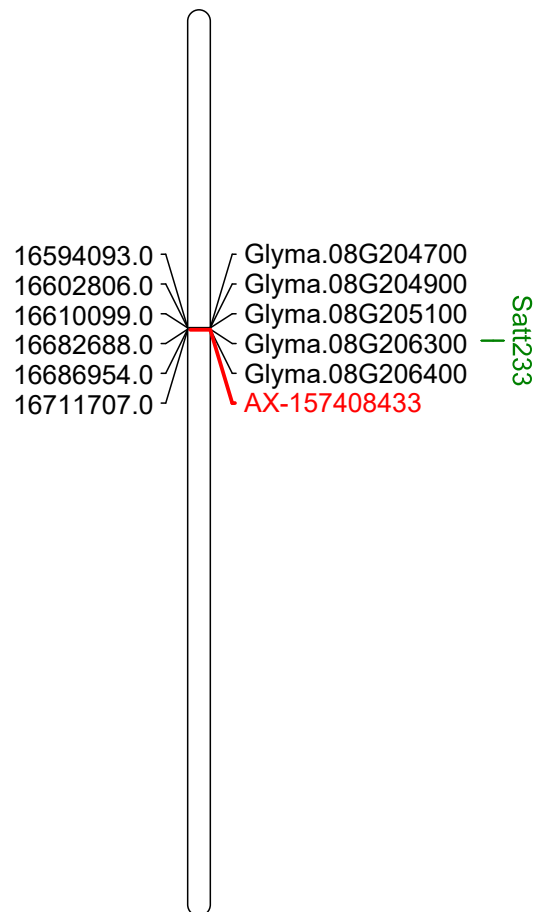

chr17

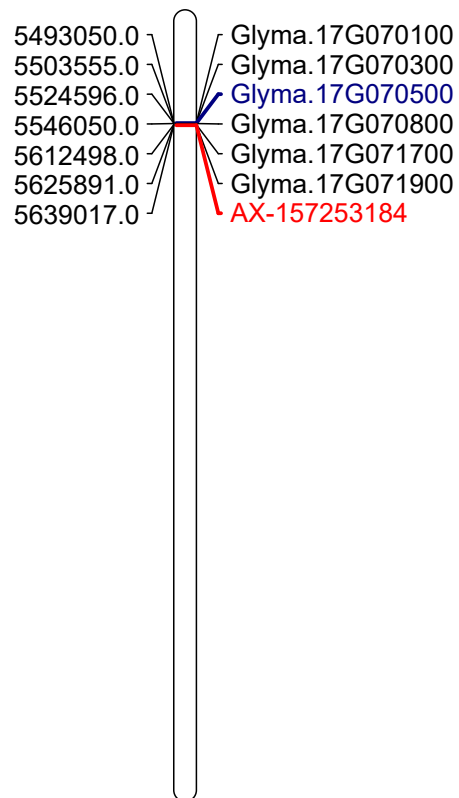

chr20

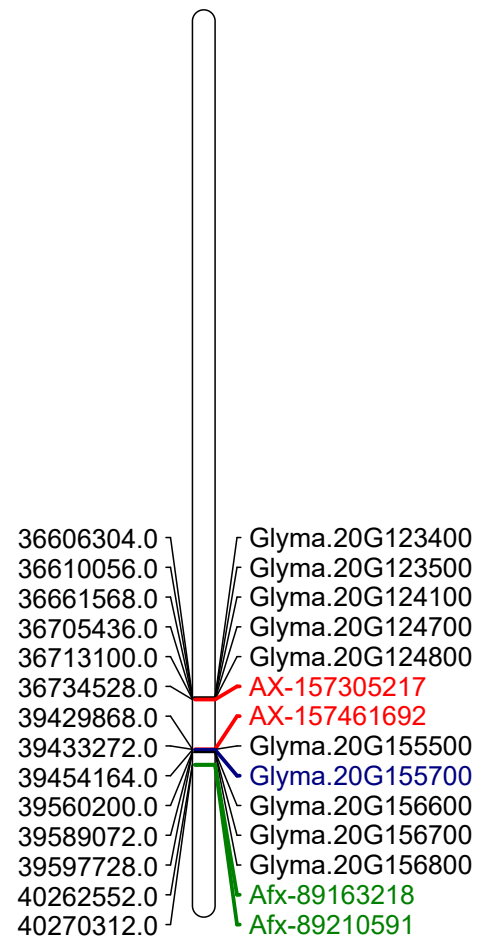

Supplement: Supplementary file 1 [file plants-15-02106-s001.zip › Figure S2.pdf]
